# Nursing Information Literacy, Evidence‐Based Nursing Competency, and Clinical Innovative Behavior Among Newly Employed Master’s‐Prepared Nurses: A Multicenter Cross‐Sectional Study

**DOI:** 10.1155/jonm/5745891

**Published:** 2026-09-24

**Authors:** Fang Zou, Weichen Mao, Caini Song, Zhen Luo, Libo Yao

**Affiliations:** ^1^ Minimally Invasive Surgery Center, The First Hospital of Changsha, Changsha, Hunan, China, cssdyyy.com; ^2^ Interventional Radiology Department, Torch Developmentzone People’s Hospital, Zhongshan, Guangdong, China; ^3^ School of Nursing, Ningxia Medical University, Yinchuan, Ningxia, China, nxmu.edu.cn; ^4^ School of Nursing, Hunan Normal University, Changsha, Hunan, China, hunnu.edu.cn

**Keywords:** clinical innovative behavior, conservation of resources theory, cumulative publication output, evidence-based nursing competency, nursing information literacy

## Abstract

**Objective:**

Guided by the conservation of resources theory, this study examined the associations of nursing information literacy and evidence‐based nursing competency with clinical innovative behavior among newly employed master’s‐prepared nurses and further explored the additional contribution of cumulative publication output.

**Methods:**

A multicenter cross‐sectional design was employed in Hunan Province, China. Anonymous online surveys were conducted using a general information questionnaire, the Chinese version of the Nursing Information Literacy Scale, the Evidence‐Based Nursing Competency Scale, and the Nurse Innovative Behavior Scale. Data analysis was performed using SPSS 27.0 software, encompassing descriptive statistics, independent‐samples *t* tests, one‐way ANOVA, Pearson correlation analysis, and hierarchical multiple linear regression analysis, along with sensitivity analyses.

**Results:**

The mean clinical innovative behavior score was 32.65 ± 8.08. Clinical innovative behavior was positively correlated with nursing information literacy (*r* = 0.783, *p* < 0.01) and evidence‐based nursing competency (*r* = 0.616, *p* < 0.01). The final regression model explained 64.1% of the variance (adjusted *R* ^2^ = 0.626). After adjustment for work experience, work pressure, and cumulative publication output, nursing information literacy (*β* = 0.680, *p* < 0.001) and evidence‐based nursing competency (*β* = 0.144, *p* = 0.002) remained positively associated with clinical innovative behavior. Adding cumulative publication output increased the explained variance by 0.9 percentage points, but this increment was not statistically significant (Δ*R* ^2^ = 0.009, *p* = 0.056). Sensitivity analyses showed that the findings for nursing information literacy and evidence‐based nursing competency were generally stable.

**Conclusions:**

Nursing information literacy and evidence‐based nursing competency were positively associated with clinical innovative behavior among newly employed master’s‐prepared nurses. Nursing managers may consider strengthening information literacy and evidence‐based practice training and providing mentoring, protected time, and resource support. The overall contribution of cumulative publication output was not statistically significant in the primary adjusted model, and publication‐related findings were model‐dependent and exploratory.

**Implication for Nursing Management:**

Nursing managers may consider incorporating information retrieval, literature appraisal, evidence synthesis, and evidence translation into orientation programs and continuing professional development, while also providing protected time and resources at the organizational level and adopting multidimensional performance evaluations.

## 1. Introduction

Innovation is a core driver for the nursing discipline to address global healthcare challenges and enhance the quality of care [1]. For newly employed master’s‐prepared nurses—a group endowed with both research literacy and clinical practice potential—the stimulation and sustainability of their innovative behavior are crucial factors for realizing personal professional value and driving practical change [2]. However, during the initial stage of occupational transition, newly employed master’s‐prepared nurses may simultaneously encounter clinical adaptation demands and expectations for research and academic development [2, 3].

According to the conservation of resources (COR) theory, individuals strive to obtain, retain, and protect their valuable personal resources (e.g., time, energy, and cognitive attention) [4]. Newly employed master’s‐prepared nurses, during this transitional period, confront a typical resource allocation dilemma, necessitating the investment of finite resources into both clinical adaptation and academic tasks simultaneously [2, 3]. The theory posits that under conditions of fixed total resources, intensive resource investment in one domain may occur at the expense of others. Therefore, consistently channeling substantial cognitive and temporal resources into academic publication activities may involve resource demands and reflect the broader academic‐development context. A question warranting examination is whether cumulative academic output is associated with clinical innovative behavior during the early‐career stage, when nurses are simultaneously adapting to clinical and academic demands.

Nevertheless, nursing information literacy and evidence‐based nursing competency are important competencies for retrieving, appraising, and applying evidence in clinical practice [5, 6]. The former enables nurses to efficiently access and evaluate information, while the latter ensures the application of best evidence to clinical practice. Previous studies have reported positive associations of nursing information literacy and evidence‐based nursing competency with innovative behavior [7–9]. Yet they commonly suffer from two limitations: First, few have explored the joint associations of these competencies and academic productivity‐related factors on innovative behavior from a theoretical perspective of resource constraints. Second, most studies treat academic output as a positive factor or an outcome variable, lacking examination of the potential tension between it and clinical practice innovation from a resource‐allocation perspective.

Therefore, grounded in the COR theory, this multicenter cross‐sectional study examined clinical innovative behavior among newly employed master’s‐prepared nurses during the early stage of their transition into clinical practice. The specific objectives include: (1) to describe the current level of innovative behavior in this population; (2) to examine the associations of nursing information literacy and evidence‐based nursing competency with clinical innovative behavior after adjustment for relevant covariates; and (3) to explore whether cumulative publication output provided additional explanatory information regarding clinical innovative behavior beyond these competency‐related resources. The third objective was exploratory because cumulative publication output may reflect multiple academic and organizational factors and does not directly measure current resource investment. The findings may inform professional development and organizational support for newly employed master’s‐prepared nurses.

## 2. Methods

### 2.1. Study Participants

This study was a multicenter cross‐sectional survey. Using convenience sampling, newly employed master’s‐prepared nurses were recruited from 20 tertiary Grade A general hospitals in Hunan Province, China, between March 2025 and October 2025.

Inclusion criteria were: (1) being a newly employed master’s‐prepared nurse working in a frontline position at the participating hospital; (2) total clinical work experience ≤ 2 years; and (3) providing informed consent and voluntary participation.

Exclusion criteria were: (1) being on long‐term (> 1 month) full‐time study, advanced training, or sick leave during the survey period; (2) holding positions primarily involving administrative management or dedicated research, i.e., nonclinical frontline functions.

Sample size estimation: Multiple linear regression was the primary analysis in this study. According to Green’s empirical rule for sample size estimation in multiple linear regression [10], *N* ≥ 50 + 8 m, where *m* represents the number of independent variables planned for inclusion in the regression model. At the study design stage, 26 independent variables were planned for inclusion, primarily including sociodemographic characteristics, nursing information literacy, and evidence‐based nursing competency. The minimum required valid sample size was therefore 258 participants. Allowing for a 10% rate of invalid questionnaires, at least 287 participants were required; therefore, the target sample size was set at 300. A total of 342 completed questionnaires were submitted, of which 12 were excluded because they contained identical responses across all scale items. Consequently, 330 valid questionnaires were included in the analysis, yielding a valid questionnaire rate of 96.5%. None of the 330 valid questionnaires contained item‐level missing data; therefore, no imputation or other missing‐data procedures were required.

### 2.2. Instruments

#### 2.2.1. General Information Questionnaire

Developed by the research team, it included age, gender, marital status, work experience, professional title, employment type, English proficiency, monthly income, department, frequency of literature review, perceived work pressure, and cumulative publication output. The cumulative publication output was explicitly defined as: the total number of original research papers formally published in academic journals as first author or corresponding author since commencing their master’s‐degree program in nursing.

#### 2.2.2. Nursing Information Literacy Scale

The original scale was developed by Jo and Ha in 2019 [11]. The Chinese version was translated and culturally adapted by Zou et al. [12], members of the present research team, through forward translation, synthesis, back‐translation, expert review, and pilot testing. The scale comprises 7 dimensions and 27 items: Problem Identification Ability (items 1–4), Identifying Potential Information Sources Ability (items 5–9), Information Retrieval Ability (items 10–13), Information Evaluation Ability (items 14–18), Information Acquisition and Management Ability (items 19–22), Information Utilization Ability (items 23–25), and Information Integration Ability (items 26–27). It uses a 5‐point Likert scale, scored from 1 (never) to 5 (always), with a total score range of 27–135. Higher scores indicate stronger nursing information literacy competency. The total Cronbach’s *α* coefficient was 0.927 in the previous validation study and 0.951 in the present sample.

#### 2.2.3. Evidence‐Based Nursing Competency Scale

Developed by Wang et al. [13], the scale comprises 23 items across four dimensions: evidence retrieval and appraisal, evidence integration, evidence dissemination, situational assessment and evidence application. It uses a 5‐point Likert scale, scored from 0 (unclear) to 4 (fully compliant), with a total score range of 0–92. Higher total scores indicate stronger evidence‐based nursing competency. The total Cronbach’s *α* coefficient was 0.951 in the previous validation study and 0.977 in the present sample.

#### 2.2.4. Nurse Innovative Behavior Scale

Developed by Bao et al. [14], it includes three dimensions (idea generation, seeking support, idea implementation) with a total of 10 items. It uses a 5‐point Likert scale, scored from 1 (never) to 5 (frequently), with a total score range of 10–50. Higher scores indicate more prominent innovative behavior. The total Cronbach’s *α* coefficient was 0.879 in the previous validation study and 0.881 in the present sample.

### 2.3. Data Collection

An anonymous online survey was conducted via a professional questionnaire platform. The study protocol was approved by the Ethics Committee of Hunan Normal University (No. 2024‐411). The first page of the questionnaire contained a detailed electronic informed consent form; participants could only proceed to the formal survey after checking “Agree”. All questions were set as mandatory, and the questionnaire could only be submitted upon completion of all items. Each IP address was restricted to a single submission. Questionnaires with identical answers across all items or containing obvious logical errors were excluded. The English version of the questionnaire is provided in Supporting Appendix S1.

### 2.4. Statistical Analysis

Data analysis was performed using SPSS 27.0 software. Categorical variables were described using frequencies and percentages, while continuous variables were described using means and standard deviations. Comparisons of innovative behavior scores between two groups were performed using an independent‐samples *t* test; comparisons among three or more groups were performed using one‐way analysis of variance or Welch’s analysis of variance, depending on the homogeneity of variances, with post hoc comparisons conducted using the Tukey or Games–Howell method, respectively. Pearson’s correlation analysis was used to assess the correlations among nursing information literacy, evidence‐based nursing competency, and innovative behavior.

Hierarchical multiple linear regression analysis was performed with the total innovative behavior score as the dependent variable. Continuous variables were entered into the model using their actual scores, while categorical variables were dummy coded; the specific coding scheme is shown in Table 1. The coding of additional variables included in the expanded‐covariate sensitivity analysis is shown in Supporting Table S1. Although work experience, work pressure, and cumulative publication output were ordinal categorical variables, they were treated as categorical variables and dummy coded because a linear relationship between their category levels and innovative behavior could not be assumed. Covariates were determined comprehensively based on the COR theory, previous studies, potential confounding relationships, and multicollinearity. Univariate analyses were used solely for descriptive and exploratory comparisons and were not the sole basis for covariate selection. Model 1 included work experience and work pressure; Model 2 added the total nursing information literacy and evidence‐based nursing competency scores to Model 1; and Model 3 further included cumulative publication output. Variables in each block were entered using the forced‐entry method. Given the potential information overlap between monthly income and work experience and the conceptual overlap between frequency of literature reading and nursing information literacy, neither variable was included in the primary model.

**TABLE 1:** Variable coding table for multiple linear regression analysis.

Variable	Variable type	Assignment method
Clinical innovative behavior	Continuous variable	Total score on the Nurse Innovative Behavior Scale, entered as a continuous score
Nursing information literacy	Continuous variable	Total score on the Chinese version of the Nursing Information Literacy Scale, included as the actual score
Evidence‐based nursing competency	Continuous variable	Total score on the Evidence‐Based Nursing Competency Scale, included as the actual score
Work experience	Categorical variable	With 12–24 months as the reference group, four dummy variables are set: < 3 months = 1, otherwise = 0; 3–< 6 months = 1, otherwise = 0; 6–< 9 months = 1, otherwise = 0; 9–< 12 months = 1, otherwise = 0
Work pressure	Categorical variable	With ‘moderate’ as the reference group, four dummy variables are defined: Very Low = 1, otherwise = 0; Relatively Low = 1, otherwise = 0; Relatively High = 1, otherwise = 0; Very High = 1, otherwise = 0
Cumulative publication output	Categorical variable	Using 1–2 papers as the reference group, three dummy variables are defined: 0 papers = 1, otherwise = 0; 3–5 papers = 1, otherwise = 0; ≥ 6 papers = 1, otherwise = 0

*Note:* Multiclass categorical variables are coded using dummy variables; the reference group is assigned a value of 0 for the corresponding dummy variables.

Multicollinearity was assessed using the variance inflation factor and tolerance, while the independence of residuals was evaluated using the Durbin–Watson statistic. Residual normality, linearity, and homoscedasticity were assessed using residual histograms, normal P–P plots, and scatterplots of standardized residuals against standardized predicted values. Standardized residuals, Cook’s distance, and leverage values were examined to identify outliers or influential observations. To assess the robustness of the results, two sensitivity analyses were conducted. First, the regression analysis was repeated after excluding the eight participants who had published six or more papers. Second, gender, marital status, professional title, employment type, English proficiency, and department were additionally adjusted for in an expanded‐covariate sensitivity analysis based on the primary model.

Harman’s single‐factor test was used to assess common method bias. All 60 items from the three scales were simultaneously entered into an unrotated principal component analysis, with eigenvalues greater than 1 used as the criterion for factor extraction. A first‐factor variance contribution of less than 50% was used as the reference criterion for indicating no serious single‐factor common method bias. All tests were two‐tailed, and *p* < 0.05 was considered statistically significant.

## 3. Results

### 3.1. Sample Characteristics and Univariate Analysis of Innovative Behavior

Participants’ ages ranged from 24 to 30 years, with females comprising 88.2% (*n* = 291). The majority of participants (201 individuals, 60.9%) had work experience between 12 and 24 months.

Univariate analysis revealed that marital status, work experience, monthly income, frequency of literature review, work pressure, and cumulative publication output were associated with the total innovative behavior score (all *p* < 0.05). However, there were no statistically significant differences in clinical innovative behavior scores based on gender, professional title, employment type, English proficiency, or department affiliation (all *p* > 0.05), as shown in Table 2. The between‐group comparison of cumulative publication output constitutes an unadjusted descriptive analysis; this study did not formally test the nonlinear relationship between this variable and clinical innovative behavior.

**TABLE 2:** Sample characteristics and univariate analysis of innovative behavior scores (*n* = 330).

Variable	Category	*n*	Clinical innovative behavior score (mean ± SD)	Statistic	*p*	Post hoc comparison
Gender	Male	39	32.36 ± 7.71	−0.241^a^	0.810	—
	Female	291	32.69 ± 8.14			

Marital status	Married	161	33.88 ± 7.79	2.714^a^	0.007	—
	Unmarried	169	31.49 ± 8.19			

Work experience (months)	< 3①	14	24.29 ± 7.35	10.667^b^	< 0.001	①, ②, ③ < ④, ⑤
	3–< 6②	18	26.61 ± 8.40			
	6–< 9③	22	28.23 ± 7.70			
	9–< 12④	75	34.48 ± 7.12			
	12–24⑤	201	33.58 ± 7.77			

Professional title	Junior‐level nurse title	45	32.09 ± 7.20	−0.502^a^	0.616	—
	Registered nurse title	285	32.74 ± 8.21			

Employment type	Permanent staff	90	32.99 ± 7.62	0.464^a^	0.643	—
	Contract staff	240	32.53 ± 8.25			

English proficiency	CET‐4	196	32.66 ± 8.18	0.018^a^	0.986	—
	CET‐6	134	32.64 ± 7.95			

Monthly income (CNY)	< 4000①	32	25.59 ± 7.92	15.713^b^	< 0.001	① < ②, ③
	4000–7000②	254	33.67 ± 7.81			
	> 7000③	44	31.91 ± 7.18			

Department	General ward	130	32.22 ± 8.41	0.547^c^	0.580	—
	ED, ICU, OR	110	33.34 ± 8.75			
	Outpatient clinic	90	32.43 ± 6.63			

Frequency of literature review	Never①	14	25.79 ± 5.04	15.394^c^	< 0.001	① < ②, ③; ①, ②, ③ < ④
	Rarely②	66	30.35 ± 5.92			
	Occasionally③	160	32.10 ± 7.61			
	Frequently④	90	36.39 ± 9.07			

Work pressure	Very low①	15	30.07 ± 6.39	5.018^c^	0.001	② < ④, ⑤
	Relatively low②	54	29.91 ± 5.93			
	Moderate③	139	32.03 ± 7.63			
	Relatively high④	73	34.88 ± 9.23			
	Very high⑤	49	34.92 ± 8.75			

Cumulative publication output	0①	33	22.09 ± 2.24	141.658^c^	< 0.001	① < ②, ③; group ④ did not differ significantly from any of the other groups.
	1–2②	248	33.59 ± 7.86			
	3–5③	41	36.12 ± 5.46			
	≥ 6④	8	29.38 ± 7.29			

*Note:* Professional title refers to the Chinese nursing professional‐title classification and does not indicate nurse practitioner status. As work experience and monthly income met the homogeneity‐of‐variance assumption, one‐way ANOVA and Tukey’s post hoc test were used; as department affiliation, frequency of literature review, work pressure, and cumulative publication output did not satisfy the assumption of homogeneity of variances, Welch’s ANOVA and the Games–Howell post hoc test were used. The numbers indicate the groups corresponding to the post hoc comparisons; “<” indicates that the clinical innovative behavior score of the group preceding it is lower than that of the group following it, and the difference is statistically significant. All tests were two‐tailed.

^a^An independent‐samples *t* test.

^b^One‐way analysis of variance (ANOVA).

^c^Welch’s ANOVA.

### 3.2. Correlations Among Major Variables

Pearson correlation analysis showed that clinical innovative behavior was positively correlated with nursing information literacy (*r* = 0.783, *p* < 0.01) and evidence‐based nursing competency (*r* = 0.616, *p* < 0.01). Nursing information literacy was also positively correlated with evidence‐based nursing competency (*r* = 0.677, *p* < 0.01; Table 3).

**TABLE 3:** Correlation analysis of major variables (*n* = 330).

Item	1	2	3
1 Clinical innovative behavior	1		
2 Nursing information literacy	0.783^∗∗^	1	
3 Evidence‐based nursing competency	0.616^∗∗^	0.677^∗∗^	1

^∗∗^ *p* < 0.01, two‐tailed.

### 3.3. Multiple Linear Regression Analysis of Factors Associated With Innovative Behavior

Model 1, which included work experience and work pressure, explained 17.0% of the variance in innovative behavior scores (*R* ^2^ = 0.170, *p* < 0.001). Model 2, which further included nursing information literacy and evidence‐based nursing competency, explained an additional 46.2% of the variance (Δ*R* ^2^ = 0.462, *p* < 0.001). Model 3, which further included cumulative publication output, explained an additional 0.9% of the variance; however, this increase did not reach statistical significance (Δ*R* ^2^ = 0.009, *p* = 0.056). The final model was statistically significant overall (*F*(13, 316) = 43.357, *p* < 0.001), explaining 64.1% of the variance in innovative behavior scores, with an adjusted *R* ^2^ of 0.626.

The variable coding table is shown in Table 1. After adjusting for work experience, work pressure, and cumulative publication output, nursing information literacy (*B* = 0.263, *β* = 0.680, 95% CI: 0.221–0.305, *p* < 0.001) and evidence‐based nursing competency (*B* = 0.054, *β* = 0.144, 95% CI: 0.019–0.088, *p* = 0.002) were both positively associated with innovative behavior. The overall incremental test for the variable cumulative publication output did not reach statistical significance (see Tables 4 and 5). Regression diagnostics revealed no obvious multicollinearity, and the assumptions of independence, normality, linearity, and homoscedasticity of the residuals were generally met (Durbin–Watson value = 1.944, VIF = 1.028–2.822).

**TABLE 4:** Fitting results of the hierarchical multiple linear regression model (*n* = 330).

Model	Variables included	*R* ^2^	Adjusted *R* ^2^	△*R* ^2^	*F*_change_	*p*_change_
Model 1	Work experience and work pressure	0.170	0.149	0.170	8.228	< 0.001
Model 2	Model 1 + nursing information literacy, evidence‐based nursing competency	0.632	0.621	0.462	200.241	< 0.001
Model 3	Model 2 + cumulative publication output	0.641	0.626	0.009	2.549	0.056

*Note:* The overall test result for the final model is *F*(13, 316) = 43.357, *p* < 0.001; the Durbin–Watson statistic is 1.944.

**TABLE 5:** Regression coefficients for the final hierarchical multiple linear regression model (*n* = 330).

Variables	*B*	SE	*β*	*t*	*p*	95% CI	VIF
Constant	6.821	1.597	—	4.271	< 0.001	3.679 to 9.964	—
Work experience (months): < 3	1.306	1.782	0.033	0.733	0.464	−2.200 to 4.812	1.745
Work experience (months): 3–< 6	1.373	1.508	0.039	0.910	0.363	−1.594 to 4.339	1.586
Work experience (months): 6–< 9	3.176	1.335	0.098	2.378	0.018	0.548 to 5.803	1.501
Work experience (months): 9–< 12	0.129	0.683	0.007	0.190	0.850	−1.213 to 1.472	1.107
Work pressure: very low	0.399	1.370	0.010	0.292	0.771	−2.296 to 3.095	1.102
Work pressure: relatively low	−0.333	0.820	−0.015	−0.407	0.685	−1.946 to 1.280	1.244
Work pressure: relatively high	−0.093	0.750	−0.005	−0.124	0.901	−1.568 to 1.381	1.309
Work pressure: very high	−0.599	0.863	−0.026	−0.694	0.488	−2.296 to 1.098	1.273
Nursing information literacy	0.263	0.021	0.680	12.434	< 0.001	0.221 to 0.305	2.631
Evidence‐based nursing competency	0.054	0.017	0.144	3.071	0.002	0.019 to 0.088	1.927
Cumulative publication output: 0	−3.369	1.522	−0.125	−2.213	0.028	−6.365 to −0.374	2.822
Cumulative publication output: 3–5	−1.108	0.876	−0.045	−1.265	0.207	−2.832 to 0.616	1.130
Cumulative publication output: ≥ 6	−1.802	1.793	−0.034	−1.005	0.316	−5.328 to 1.725	1.028

*Note:* The reference group for work experience is 12–24 months; for work pressure, the reference group is “moderate,” and for cumulative publication output, the reference group is 1–2 papers. *B* denotes the unstandardized regression coefficient, SE denotes the standard error, *β* denotes the standardized regression coefficient, 95% CI denotes the 95% confidence interval, and VIF denotes the variance inflation factor. Variables at all levels were included in the model using the forced entry method. The incremental variation in explained variance contributed by the cumulative publication output variable group was not statistically significant (Δ*R* ^2^ = 0.009, *p* = 0.056); the coefficients of its respective dummy variables are presented for exploratory purposes only.

### 3.4. Common Method Bias Test

The results of Harman’s single‐factor test showed that the KMO value was 0.964, and Bartlett’s sphericity test was statistically significant (*χ* ^2^ = 15,554.939, df = 1,770, *p* < 0.001). Unrotated principal component analysis identified a total of 10 factors with eigenvalues greater than 1. The first factor explained 42.928% of the total variance, which is below the reference threshold of 50%. This does not indicate severe common‐method bias attributable to a single factor, but the possibility of common‐method bias cannot be entirely ruled out.

### 3.5. Sensitivity Analysis

After excluding the eight participants who had published six or more papers (*n* = 322), the effect estimates for nursing information literacy (*B* = 0.266, *β* = 0.690, *p* < 0.001) and evidence‐based nursing competency (*B* = 0.051, *β* = 0.137, *p* = 0.004) were generally consistent with those in the primary analysis. In the expanded‐covariate sensitivity analysis (*n* = 330), nursing information literacy (*B* = 0.259, *β* = 0.670, *p* < 0.001) and evidence‐based nursing competency (*B* = 0.054, *β* = 0.145, *p* = 0.002) remained positively associated with clinical innovative behavior, with effect estimates similar to those in the primary analysis. The overall test for cumulative publication output did not reach the conventional significance threshold in the primary model (*p* = 0.056), but yielded *p* values of 0.034 after excluding participants with six or more publications and 0.044 in the expanded covariate‐adjusted model. Because the statistical conclusion for cumulative publication output was sensitive to sample composition and covariate adjustment, the publication‐related findings were considered model‐dependent and exploratory. The coding of the additional covariates is presented in Supporting Table S1, and summary results of the primary and sensitivity analyses are presented in Supporting Table S2.

## 4. Discussion

### 4.1. Status of Clinical Innovative Behavior Among Newly Employed Master’s‐Prepared Nurses

In this study, the mean clinical innovative behavior score among newly employed master’s‐prepared nurses was 32.65 ± 8.08, slightly higher than the scale’s theoretical midpoint of 30. This score was also higher than the score of 28.36 ± 6.25 reported among clinical nurses in China [15]. A recent multicenter study using a different measurement instrument similarly reported a moderate‐to‐high level of innovative behavior among intensive care unit nurses in China [16]. Variations across studies may reflect differences in participant characteristics, research training received during postgraduate education, clinical work environments, and measurement instruments. Overall, the findings suggest that there remains room for further improvement in clinical innovative behavior among newly employed master’s‐prepared nurses.

Unadjusted analyses indicated that clinical innovative behavior scores varied according to marital status, work experience, monthly income, frequency of consulting the literature, work pressure, and cumulative publication output. These group‐level variations provide a descriptive profile of clinical innovative behavior across individual, academic, and work‐related characteristics. The differences observed across cumulative publication output groups also warrant further investigation of the relationship between academic engagement and clinical innovation.

### 4.2. Factors Associated With Clinical Innovative Behavior Among Newly Employed Master’s‐Prepared Nurses

#### 4.2.1. Association Between Nursing Information Literacy and Clinical Innovative Behavior

Multiple linear regression analysis showed that nursing information literacy was positively associated with clinical innovative behavior among newly employed master’s‐prepared nurses (*β* = 0.680, *p* < 0.001). This finding is consistent with Wang et al. [9], who reported a positive correlation between information literacy and innovative behavior among specialist nurses. Similarly, Ge et al. [16] found that information literacy was significantly associated with innovative behavior among intensive care unit nurses. In an increasingly information‐intensive healthcare environment, the ability to retrieve, appraise, and apply relevant information may help nurses identify clinical problems, evaluate possible solutions, and translate evidence into practice [17]. This ability may be particularly relevant to newly employed master’s‐prepared nurses as they transition from postgraduate education to clinical practice.

From the perspective of the COR theory, nursing information literacy may be conceptualized as a personal resource that nurses can draw upon in evidence‐informed problem‐solving [4]. This interpretation is consistent with the application of COR theory to nurses’ innovative behavior [18]. By supporting access to and appraisal of relevant evidence, nursing information literacy may assist nurses in developing and implementing clinical improvement strategies. These findings highlight the potential value of incorporating information literacy development into the professional support provided to newly employed master’s‐prepared nurses.

#### 4.2.2. Association Between Evidence‐Based Nursing Competency and Clinical Innovative Behavior

Evidence‐based nursing competency remained positively associated with clinical innovative behavior among newly employed master’s‐prepared nurses after adjustment for nursing information literacy and other covariates (*β* = 0.144, *p* = 0.002). This finding is consistent with Wang et al. [9], who reported that evidence‐based nursing competency was associated with innovative behavior and mediated the relationship between information literacy and innovative behavior among specialist nurses. The present study adds evidence from newly employed master’s‐prepared nurses. Evidence‐based nursing competency involves the appraisal, integration, and application of research evidence in clinical decision‐making [17]. In the context of clinical innovation, this competency may help nurses evaluate whether a proposed improvement is scientifically supported, applicable to the target patient population, and feasible within the local clinical environment.

From the perspective of the COR theory, evidence‐based nursing competency may be conceptualized as a personal resource relevant to the evaluation and refinement of clinical improvement strategies [4]. Nursing information literacy and evidence‐based nursing competency represent related but distinct competencies: information literacy supports the identification, retrieval, and appraisal of relevant information, whereas evidence‐based nursing competency emphasizes the integration of evidence with clinical expertise and its application to practice [17]. In the adjusted model, both competencies were independently associated with clinical innovative behavior. These findings highlight the value of strengthening evidence‐based nursing competency alongside nursing information literacy in the professional development of newly employed master’s‐prepared nurses.

#### 4.2.3. Association Between Cumulative Publication Output and Clinical Innovative Behavior

In the hierarchical regression analysis, the addition of cumulative publication output increased the explained variance by 0.9% points, but this increment was not statistically significant (Δ*R* ^2^ = 0.009, *p* = 0.056). Accordingly, cumulative publication output did not significantly improve the explanatory capacity of the adjusted model.

The unadjusted group comparisons displayed a preliminary nonmonotonic pattern in clinical innovative behavior scores across cumulative publication output categories, providing a hypothesis‐generating observation for future research. As no formal test of nonlinearity was conducted and the group with six or more publications included only eight participants, this pattern remains descriptive and does not establish an inverted U‐shaped relationship.

In the present study, cumulative publication output was treated as an academic background characteristic rather than a direct measure of academic resource investment. Future studies with larger and more evenly distributed samples could formally test potential nonlinear relationships and examine how publication‐related academic engagement is associated with clinical innovative behavior from the resource‐allocation perspective of the COR theory [4].

### 4.3. Theoretical and Research Contributions

This study contributes to the literature by jointly examining nursing information literacy, evidence‐based nursing competency, and cumulative publication output in relation to clinical innovative behavior among newly employed master’s‐prepared nurses. Nursing information literacy and evidence‐based nursing competency showed generally consistent positive associations across the primary and sensitivity analyses, whereas findings related to cumulative publication output varied across analytical approaches.

From the perspective of the COR theory, nursing information literacy and evidence‐based nursing competency may be conceptualized as personal resources relevant to clinical innovation. Cumulative publication output, in contrast, represents a retrospective academic characteristic and captures a different dimension from current competency‐related resources. This distinction offers a more differentiated perspective on the factors associated with clinical innovative behavior among newly employed master’s‐prepared nurses.

### 4.4. Implications for Nursing Management

The positive associations of nursing information literacy and evidence‐based nursing competency with clinical innovative behavior highlight their relevance to the professional development of newly employed master’s‐prepared nurses. Nursing managers may consider incorporating information retrieval, literature appraisal, evidence synthesis, and evidence translation into orientation programs and continuing professional development. Case‐based learning and evidence‐based practice projects could provide opportunities to apply these competencies to clinical problems. Mentorship arrangements involving nursing managers, experienced clinical nurses, and educational partners may also support the transition into clinical practice [19].

At the organizational level, nursing managers may consider providing protected time and appropriate resources for literature retrieval, evidence appraisal, quality improvement, and innovation‐related activities. Performance evaluations could incorporate multiple dimensions, including the quality of clinical care, evidence‐based practice, teamwork, quality improvement, and innovation‐related contributions. In this study, cumulative publication output did not significantly improve the explanatory capacity of the adjusted model, and publication‐related findings varied across analytical approaches. These findings suggest that publication quantity alone may be insufficient for evaluating the clinical innovative behavior of newly employed master’s‐prepared nurses.

## 5. Limitations

This study has several limitations. First, the cross‐sectional design precludes conclusions about temporal or causal relationships among the study variables. Future longitudinal or interventional studies are needed to further examine these relationships.

Second, convenience sampling was used, and participants were recruited from 20 tertiary Grade A hospitals in Hunan Province, China, which may limit the generalizability of the findings to other regions and hospital settings. Because hospital identifiers were not retained, hospital‐level clustering could not be assessed or adjusted for. Future studies should retain hospital‐level information and use multilevel models or cluster‐adjusted methods where appropriate.

Third, cumulative publication output was analyzed as a categorical variable, and only eight participants were included in the group with six or more publications. In addition, publication‐related findings varied across the primary and sensitivity analyses. These findings should therefore be regarded as exploratory. Although covariates were selected based on theoretical considerations, previous evidence, potential confounding relationships, and multicollinearity assessment, residual confounding may remain.

Finally, the primary variables were measured concurrently using self‐report questionnaires and may be susceptible to social desirability and common method bias. Although Harman’s single‐factor test did not suggest that a single factor dominated the variance, common method bias cannot be ruled out. Future studies could incorporate objective indicators and assessments from multiple sources to further validate the findings.

## 6. Conclusion

Based on the COR theory, this study examined the relationships of nursing information literacy, evidence‐based nursing competency, and cumulative publication output with clinical innovative behavior among newly employed master’s‐prepared nurses. Clinical innovative behavior was slightly above the theoretical midpoint, and nursing information literacy and evidence‐based nursing competency were positively associated with it. The overall test for cumulative publication output did not reach statistical significance in the primary adjusted model, whereas publication‐related findings varied across analytical models. Given the cross‐sectional design, these findings do not establish causal relationships. Nursing managers may consider strengthening information literacy and evidence‐based practice development, providing mentorship, protected time, and appropriate resources, and adopting multidimensional performance evaluations rather than relying solely on publication quantity.

## Author Contributions

Fang Zou: conceptualization, data curation, and writing–original draft.

Weichen Mao: data curation and formal analysis.

Caini Song: methodology, formal analysis, and writing–review and editing.

Zhen Luo: data curation.

Libo Yao: supervision and writing–review and editing.

## Funding

This work was supported by the 2026 Nursing Psychological Science Research Program (grant no. YB2026071).

## Ethics Statement

This study adhered to the Declaration of Helsinki. This study was conducted after obtaining ethical approval from the Ethics Committee of Hunan Normal University (No. 2024‐411). Informed consent to participate was obtained from all of the participants in the study.

## Consent

Please see the Ethics statement.

## Conflicts of Interest

The authors declare no conflicts of interest.

## Supporting Information

Additional supporting information can be found online in the Supporting Information section.

## Supporting information


**Supporting Information** Supporting Appendix S1 presents the researcher‐developed General Information Questionnaire. Supporting Table S1 presents the coding of additional covariates included in the expanded‐covariate sensitivity analysis. Supporting Table S2 compares the results of the primary and sensitivity analyses.

## Data Availability

The data that support the findings of this study are available from the corresponding author upon reasonable request.
