# Supplementary material for: Nursing Information Literacy, Evidence‐Based Nursing Competency, and Clinical Innovative Behavior Among Newly Employed Master’s‐Prepared Nurses: A Multicenter Cross‐Sectional Study
Source: J Nurs Manag. 2026 Sep 24;2026:5745891. doi: 10.1155/jonm/5745891 (PMC13602810; doi:10.1155/jonm/5745891)
Supplement: Supplementary file 1 [file JONM-2026-5745891-s001.docx]

**Supplementary Appendix S1. Researcher-Developed General Information Questionnaire**

The questionnaire was administered in Chinese. This English translation is provided for readers’ reference. Please select one response for each item unless otherwise indicated.

1. Age: ______ years
2. Gender:	Male; Female
3. Marital Status:	Married; Unmarried
4. Work Experience (months): <3; 3–<6; 6–<9; 9–<12; 12–24 months
5. Professional Title:	Registered nurse title; Junior-level nurse title
6. Employment Type:	Permanent Staff; Contract Staff
7. English Proficiency:	CET-4; CET-6
8. Monthly Income (CNY): <4,000; 4,000–7,000; >7,000
9. Department:	General Ward; ED, ICU, OR; Outpatient Clinic
10. Frequency of Literature Review: Never; Rarely; Occasionally; Frequently
11. Perceived Work Pressure: Very Low; Relatively Low; Moderate; Relatively High; Very High

12. Cumulative Publication Output: 0; 1–2; 3–5; ≥6

Definition: Cumulative publication output was defined as the total number of original research papers formally published in academic journals as first author or corresponding author since commencing the master’s degree program in nursing.

**Supplementary Table S1. Coding of Additional Covariates Included in the Expanded-Covariate Sensitivity Analysis**

| Variable | Variable Type | Assignment Method |
| --- | --- | --- |
| Gender | Dichotomous variable | Female = 0, Male = 1 |
| Marital status | Dichotomous variable | Unmarried = 0, Married = 1 |
| Professional title | Dichotomous variable | Registered nurse title = 0, Junior-level nurse title = 1 |
| Employment type | Dichotomous variable | Contract Staff = 0, Permanent Staff = 1 |
| English proficiency | Dichotomous variable | CET-4 = 0, CET-6 = 1 |
| Department | Categorical variable | Using the General Ward as the reference group, two dummy variables are defined: ED, ICU, OR = 1, otherwise = 0; Outpatient Clinic = 1, otherwise = 0 |

Note: In the expanded-covariate sensitivity analysis, the coding of clinical innovative behavior, nursing information literacy, evidence-based nursing competency, work experience, work pressure, and cumulative publication output was identical to that used in the primary analysis. CET-4 refers to the College English Test Band 4, and CET-6 refers to the College English Test Band 6.

**Supplementary Table S2. Comparison of the Primary Analysis and Sensitivity Analyses**

| Analytical Model | n | Adjusted R^2^ | Nursing Information Literacy B | Nursing Information Literacy β | *P* | Evidence-Based Nursing Competency B | Evidence-Based Nursing Competency β | *P* | ΔR² for Cumulative Publication Output | Overall P for Cumulative Publication Output |
| --- | --- | --- | --- | --- | --- | --- | --- | --- | --- | --- |
| Primary analysis | 330 | 0.626 | 0.263 | 0.680 | < 0.001 | 0.054 | 0.144 | 0.002 | 0.009 | 0.056 |
| Excluding the group with ≥6 published papers | 322 | 0.628 | 0.266 | 0.690 | < 0.001 | 0.051 | 0.137 | 0.004 | 0.008 | 0.034 |
| Expanded-covariate sensitivity analysis | 330 | 0.624 | 0.259 | 0.670 | < 0.001 | 0.054 | 0.145 | 0.002 | 0.009 | 0.044 |

Note: The sensitivity analysis excluding participants who had published a cumulative total of six or more papers did not include the eight participants in this group. The expanded-covariate sensitivity analysis model further adjusted for gender, marital status, professional title, employment type, English proficiency, and department of affiliation, in addition to the variables in the primary model. The overall test for cumulative publication output varied across models; therefore, the publication-related findings were considered model-dependent and exploratory.
